# Chemical Cocktail Induces Hematopoietic Reprogramming and Expands Hematopoietic Stem/Progenitor Cells

**DOI:** 10.1002/advs.201901785

**Published:** 2019-11-11

**Authors:** Yi Zhou, Xingli Zhu, Yuting Dai, Shumin Xiong, Chuijin Wei, Pei Yu, Yuewen Tang, Liang Wu, Jianfeng Li, Dan Liu, Yanlin Wang, Zhu Chen, Sai‐Juan Chen, Jinyan Huang, Lin Cheng

**Affiliations:** ^1^ State Key Laboratory of Medical Genomics Shanghai Institute of Hematology Rui Jin Hospital affiliated to Shanghai Jiao Tong University School of Medicine Key Laboratory of Systems Biomedicine (Ministry of Education) and Collaborative Innovation Center of Systems Biomedicine Shanghai Center for Systems Biomedicine Shanghai Jiao Tong University Shanghai 200025 China; ^2^ School of Life Sciences and Biotechnology Shanghai Jiao Tong University 200025 Shanghai China; ^3^ Department of Orthopaedics Rui Jin Hospital affiliated to Shanghai Jiao Tong University School of Medicine Shanghai 200025 China; ^4^ Prenatal Diagnosis Center International Peace Maternity and Child Health Hospital affiliated to Shanghai Jiao Tong University School of Medicine Shanghai 200030 China; ^5^ Pôle de Recherches Sino‐Français en Science du Vivant et Génomique Laboratory of Molecular Pathology Rui‐Jin Hospital Shanghai Jiao Tong University School of Medicine Shanghai 200025 China

**Keywords:** cell expansion, cell fate change, cell reprogramming, chemical cocktails, hematopoietic stem/progenitor cells

## Abstract

Generation of hematopoietic stem/progenitor cells (HSPCs) via cell expansion or cell reprogramming has been widely achieved by overexpression of transcription factors. Herein, it is reported that without introducing exogenous genes, mouse fibroblasts can be reprogrammed into hemogenic cells based on lineage tracing analysis, which further develop into hematopoietic cells, by treatment of cocktails of chemical compounds. The chemical cocktails also reprogram differentiated hematopoietic cells back into HSPC‐like cells. Most importantly, the chemical cocktails enabling hematopoietic reprogramming robustly promote HSPC proliferation ex vivo. The expanded HSPCs acquire enhanced capacity of hematopoietic reconstruction in vivo. Single‐cell sequencing analysis verifies the expansion of HSPCs and the cell reprogramming toward potential generation of HSPCs at the same time by the chemical cocktail treatment. Thus, the proof‐of‐concept findings not only demonstrate that hematopoietic reprogramming can be achieved by chemical compounds but also provide a promising strategy for acquisition of HSPCs by chemical cocktail‐enabled double effects.

## Graphical abstract

A chemical cocktail is used to convert differentiated hematopoietic cells into hematopoietic stem/progenitor cell (HSPC)‐like cells. The cocktail can also robustly promote HSPC expansion ex vivo. This study is the proof‐of‐concept that hematopoietic reprogramming can be induced only by chemical compounds without introduction of exogenous genes, and generation of HSPCs can be achieved simultaneously via two diverse directions: expansion and reprogramming.

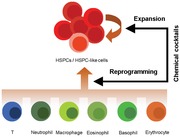

## 1 Introduction

Hematopoietic stem/progenitor cells (HSPCs) hold great promise for treating hematopoietic system diseases. However, limited resources and cell number of HSPCs hurdle the application of these cells in clinic. Expansion of HSPCs has been reported via exogenous expression of key transcription factors.^1^ It is also found that the transcription factors implicated in HSPC expansion and development can also reprogram somatic cells into HSPCs in vitro.^2^, ^3^, ^4^, ^5^, ^6^ These expanding and advanced reprogramming strategies might yield inexhaustible hematopoietic cells for cell therapy. However, the conventional methods are based on viruses‐mediated expression of exogenous genes, which requires genetic manipulation and is bare to be translated into clinical application due to safety concerns.

Chemical compounds with compelling advantages are potential alternative for cell expansion and manipulating cell fate change. Many reports have demonstrated that combination of chemical compounds can completely replace transcription factors to convert somatic cells into desirable cells, including induced pluripotent stem cells,^7^, ^8^, ^9^, ^10^ neural stem/progenitor cells,^11^, ^12^, ^13^ endodermal progenitors,^14^ neurons,^15^, ^16^, ^17^ and cardiomyocytes.^18^, ^19^ Although HSPCs or hematopoietic cells can be induced from non‐ or differentiated‐hematopoietic cells via transcription factors, whether these cells can be induced by chemical compounds remains unknown.

In this paper, we show that combination of chemical compounds not only converts mouse fibroblasts into cells with hemogenic potential and differentiated hematopoietic cells into HSPC‐like cells, but also promotes HSPC expansion ex vivo. This is the first proof of concept that process of hematopoiesis and its reverse process can be mimicked in vitro by defined cocktails of chemical compounds.

## 2 Results

### 2.1 Chemical Cocktails Activate Hematopoietic Program in Mouse Fibroblasts

Previous studies demonstrated that Sox2 played an important role during early regenerative responses of hematopoietic system and overexpression of single transcription factor Sox2 can reprogram fibroblasts toward CD34 positive (CD34^+^) cells with hematopoietic potential.^20^ We and others have found diverse cocktails of chemical compounds enabling cell fate change, which was mainly dependent on Sox2 activation.^11^, ^12^, ^21^ All these together make us to hypothesize that the identified chemical cocktails activating Sox2 might enable fibroblasts with hematopoietic potential. To prove this hypothesis, we first treated mouse embryonic fibroblasts with diverse compounds, including chemical cocktail #1 (CC1) (Valproic acid (VPA), CHIR99021, and Repsox), chemical cocktail #2 (CC2) (CHIR99021, LDN193189, A83‐01, Hh‐Ag1.5, Vitamin C, SMER28, RG108, and Parnate), and chemical cocktail #3 (CC3) (E616452, CHIR99021, Tranylcypromine, and Forskolin). Quantitative real‐time PCR (qRT‐PCR) analysis demonstrated that all three chemical cocktails can activate Sox2. Expression level of Sox2 was much higher in both CC1 and CC2 treated groups than that in CC3 treated one at different time points. Consistent with this result, CD34 expression is significantly activated by CC1 and CC2, but not by CC3 (Figure S1a, Supporting Information). Based on these primary data, we focus on the former two chemical cocktails for subsequent hematopoietic cell induction.

**Figure 1:**
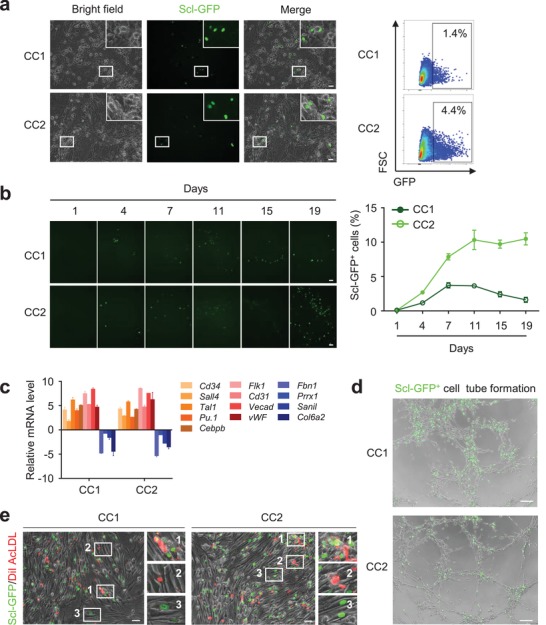
Induction of hemogenic cells from mouse fibroblasts by chemical cocktails. a) Generation of Scl‐GFP^+^ cells from Scl‐GFP^−^ fibroblasts treated with chemical cocktails CC1 or CC2 for 5 d. Representative figures (left). Fluorescence‐activated cell sorting (FACS) analysis (right). b) Detection of Scl‐GFP^+^ cell generation from Scl‐GFP^−^ fibroblasts treated with chemical cocktails CC1 or CC2 on different days. Representative figures (left). Quantification of Scl‐GFP^+^ cell percentage analyzed by FACS (right). c) qRT‐PCR analysis of hemogenic genes and fibroblast genes. All data are normalized to that of control. d) Tube formation assay for CC1 or CC2 induced Scl‐GFP^+^ cells cultured in Matrigel for 4 h. e) Induced Scl‐GFP^+^ cells by CC1 or CC2 were further cultured as adherent then stained by DiI AcLDL dye (Red). Scale bar, 50 µm.

Among the transcription factors reported previously to induce hematopoietic cells from somatic cells, Scl is the key factor included in most recipes. The expression of *Scl* is highly enriched in endothelial cells and hematopoietic cells with stem/progenitor properties.^22^ Thus we utilized a double‐transgenic mouse, Scl‐tTA × TetO‐H2BGFP (called Scl‐GFP), as a lineage tracing system in our study. Green fluorescent protein (GFP) is specifically expressed under control of *Scl* promoter, which is considered as reporter when hemogenic fate is acquired. To avoid contamination of hematopoietic cells and GFP^+^ cell, CD45^+^ cells and GFP^+^ cells were removed from primary fibroblasts via cell sorting prior to chemical induction (Figure S1b, Supporting Information). Remaining CD45^−^Scl‐GFP^−^ fibroblasts were used as initial cells for further inducing assays. As demonstrated in schematic model (Figure S1c, Supporting Information), starting fibroblasts were treated with chemical cocktails in DMEM for two days. Then the culture medium was switched into HSPC maintaining medium M5300 including cytokines stem cell factor (Scf), FMS‐like tyrosine kinase 3 ligand (Flt3l), interleukin‐3 (IL‐3), and interleukin‐6 (IL6). Scl‐GFP^+^ cells were observed obviously and individually in both CC1 and CC2 treated fibroblasts (**Figure** **1**a). These Scl‐GFP^+^ cells emerged as early as four days after chemical treatment and continued to increase over time. Comparatively, cell reprogramming efficiency was higher in CC2 than that in CC1 (Figure 1b), which was calculated by the percentage of Scl‐GFP^+^ cells among the total cells.

### 2.2 Chemical Cocktail Induced Scl‐GFP**^+^** Cells Acquire Hemogenic Potential

To characterize transcriptional profile of induced Scl‐GFP^+^ cells, we conducted mRNA sequence of initial CD45^−^Scl‐GFP^−^ cells, CC1 induced Scl‐GFP^+^ cells on day 11 and day 19, CC2 induced Scl‐GFP^+^ cells on day 8 and day 13, and primary Scl‐GFP^+^ cells isolated from bone marrow (BM). The primary BM Scl‐GFP^+^ cells mainly consisted of Lin^−^Sca1^+^cKit^+^ (LSK) HSPCs. Unsupervised hierarchical clustering analysis demonstrated that chemical induced Scl‐GFP^+^ cells were not yet closer to primary BM Scl‐GFP^+^ cells. However, principle component analysis showed that the chemical treatments still promoted fibroblast conversion toward BM Scl‐GFP^+^ cells (Figure S1d, Supporting Information). Expression profiles showed minor differences in Scl‐GFP^+^ cells generated on different days by the same chemical cocktail treatment, but still showed major differences in Scl‐GFP^+^ cells generated by these two chemical cocktails with distinct components. Therefore, except for the key transcription factor Sox2 being activated for initial cell reprogramming as we proposed, additional factors affecting the reprogramming process might also be activated. Expression of fibroblast‐related genes enriched in CD45^−^GFP^−^ cells such as *Fbn1*, *Prrx1*, *Snail1*, and *Col6a2* decreased in induced Scl‐GFP^+^ cells. These chemical induced Scl‐GFP^+^ cells highly expressed hematopoietic markers such as *CD41*, *Sox17*, *PU.1*, *CEBPa*, *CEBPb* and *Tal1*, and endothelial or endothelial progenitor markers such as *Flk1*, *CD31*, *Vecad*, and *vWF*. Expression of these genes was further verified by qRT‐PCR analysis (Figure 1c).

To better understand the biological characteristics of these induced Scl‐GFP^+^ cells, we checked on cell morphology and cell function. Among the chemical cocktail treated cells, most Scl‐GFP^+^ cells appeared adherent and a few of these cells appeared semiadherent and suspended. To further characterize these chemical induced Scl‐GFP^+^ cells, expression of hemogenic progenitor cell makers including CD133, CD144, and Sca‐1 were examined. After 11 d, 73.6% of CC1 induced Scl‐GFP^+^ cells were Sca‐1^+^, 1.5% were CD133^+^, and 6.1% were CD144^+^. 9.1% of CC2 induced Scl‐GFP^+^ cells were Sca‐1^+^, 3% were CD144^+^, and 3.2% were CD133^+^ (Figure S1e, Supporting Information). Tube formation assay demonstrated that these induced Scl‐GFP^+^ cells can form vessel‐like structures in vitro (Figure 1d). Besides, further cultured the induced cells in medium containing vascular endothelial growth factor, a few Scl‐GFP^+^ cells and Scl‐GFP^+^‐derived cells acquired ability of uptaking low‐density lipoprotein (Figure 1e). Both assays show that the induced Scl‐GFP^+^ cells were characterized by hemogenic cell‐like properties.

We next decided to investigate optimal conditions for the chemical induced hemogenic cell generation. Addition of E4EC, endothelial cells expressing adenoviral E4ORF1 gene, or Matrigel significantly enhanced the generating efficiency of hemogenic cells from fibroblasts induced by both CC1 and CC2 (Figure S1f,g, Supporting Information). Scl‐GFP^+^ cells sorted from E4EC coculture group even further developed into CD45^+^ cells, which mainly expressed monocyte/macrophage marker CD11b (Figure S1h, Supporting Information).

### 2.3 Chemical Cocktails Induce Hematopoietic Cell Reprogramming

**Figure 2:**
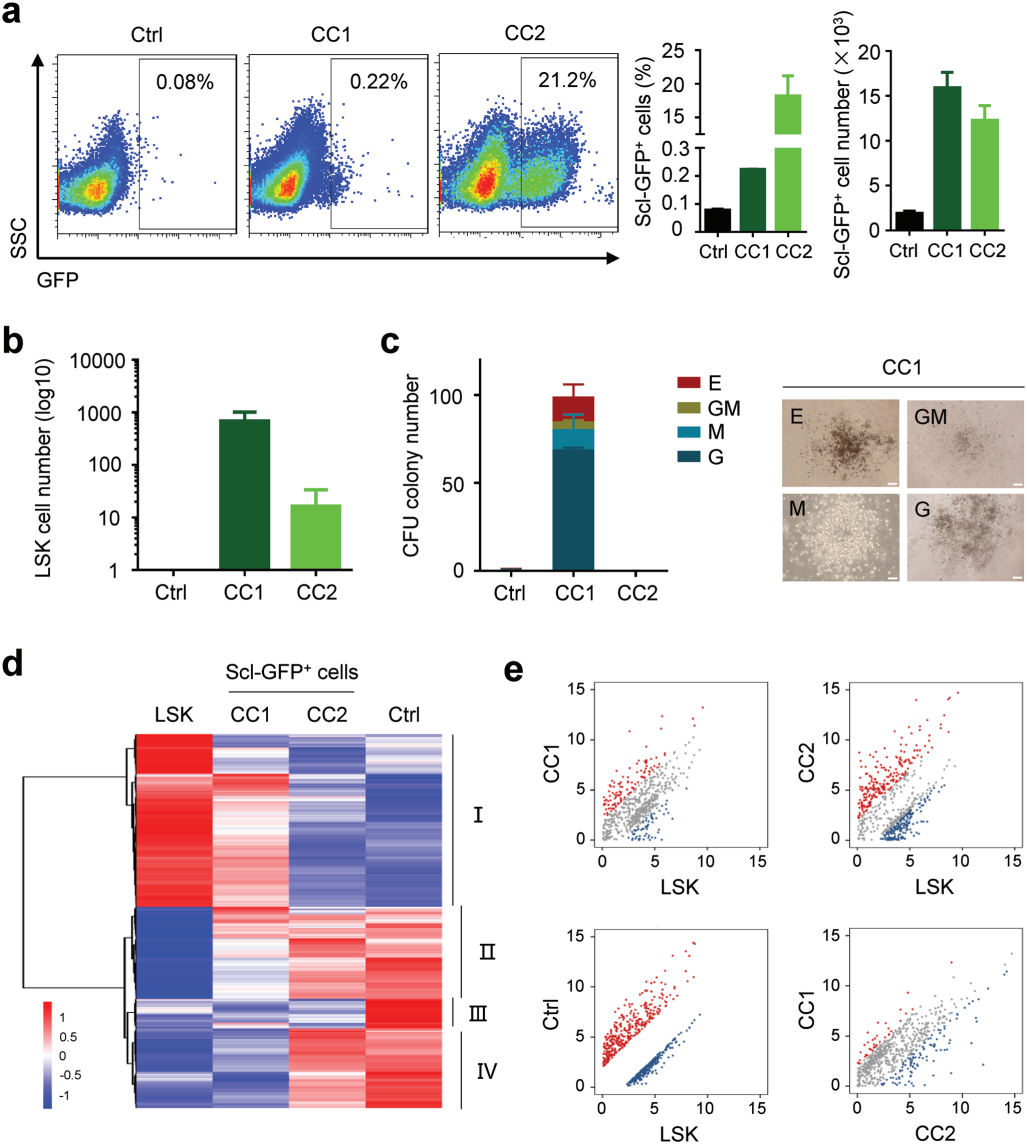
Chemical cocktails enable committed hematopoietic cell reprogram back. a) Bone marrow derived Scl‐GFP^−^ hematopoietic cells were reprogrammed into Scl‐GFP^+^ cells by the treatment of chemical cocktails on day 7. Representative data of FACS analysis (left). Quantification of Scl‐GFP^+^ cell percentage (middle). Quantification of Scl‐GFP^+^ cell number (right). b) Detection of LSK cell numbers in chemical induced Scl‐GFP^+^ cells. c) The chemical induced Scl‐GFP^+^ cells were cultured in M3434 for 14 d to investigate the colony formation ability. Quantify the colony numbers formed by each hematopoietic cell (left). Representative figures of the colonies formed by CC1 induced Scl‐GFP^+^ cells. Scale bar, 200 µm. d) Heatmap of genes with significance from mRNA sequence analysis data. Samples of primary LSK cells, bone marrow derived Scl‐GFP^−^ cells as ctrl, and Scl‐GFP^+^ cells derived from CC1 or CC2 treatment. Red indicates increased expression and blue represents decreased expression as compared to that in control. e) Pairwise scatter plot analysis of genes in (a). Red dots represent upregulated gene expression, blue dots represent downregulated genes, and gray dots represent no significant difference.

Comparing with transcription factors‐induced HSPC‐like cells from fibroblasts, functional HSPCs have been reprogrammed from pre/pro‐B cells, which demonstrates that reprogramming among the same lineages might generate stem/progenitor cells more efficiently than that between trans‐lineages. Following this logistics, we investigated that whether the chemical cocktails inducing hemogenic reprograming in fibroblasts could reprogram differentiated hematopoietic cells back to progenitors even to acquire stemness. Based on the above lineage tracing system, and to avoid any Scl‐GFP^+^ cell contamination in the initial cells, blood cells from Scl‐GFP mice were sorted twice to remove Scl‐GFP^+^ cells, then these double‐sorted cells were seeded in 96‐well plate and checked under fluorescence microscope to kick out Scl‐GFP^+^ cells again (Figure S2a, Supporting Information). Scl‐GFP^−^ blood mononuclear cells isolated from bone marrow were treated the chemical cocktails. One week later, Scl‐GFP^+^ cells were generated from both chemical treated groups (Figure S2b, Supporting Information). Consistent with previous data, reprogramming efficiency from Scl‐GFP^−^ blood mononuclear cells to Scl‐GFP^+^ cells was significantly higher in CC2 than that in CC1 (**Figure** **2**a). This reprogramming process was also observed in blood mononuclear cells from spleen treated with CC2 or CC1 (Figure S2c, Supporting Information). For cells from both tissues, CC1 promoted hematopoietic cell expansion more significantly than CC2. Accordingly, cell number of Scl‐GFP^+^ cells was relatively higher in CC1 than that in CC2 (Figure S2d, Supporting Information).

Remarkably, LSK cells were detected in both CC1‐ or CC2‐induced Scl‐GFP^+^ hematopoietic cells. LSK cell number was significantly higher in CC1 than that in CC2 (Figure 2b; Figure S2e, Supporting Information). Importantly, 2‐week culture of these chemical‐induced Scl‐GFP^+^ cells in methylcellulose‐based semisolid media, colonies with diverse morphologies representing colony forming unit‐erythrocyte (CFU‐E), CFU‐granulocyte and macrophage (CFU‐GM), CFU‐macrophage (CFU‐M), and CFU‐granulocyte (CFU‐G), were observed in CC1, but not in control group and CC2 (Figure 2c). mRNA sequence analysis demonstrated that both CC1 and CC2 treatment inhibited the expression of genes related to terminal differentiated hematopoietic cells, which are responsible for inflammatory bowel disease, graft‐versus‐host disease, etc. However, the genes related to cells about innate immune response were mainly inactivated in CC1, but not CC2. Importantly, HSPC‐related specific genes were also significantly activated in CC1, which was similar to that in primary LSK cells (Figure 2d; Figure S2f, Supporting Information). Scatter plot analysis showed a high degree of similarity between CC1 derived Scl‐GFP^+^ cells and primary LSK cells, and significant difference between LSK cells and ctrl one (Figure 2e).

### 2.4 Chemical Cocktail Expands HSPCs Ex Vivo

**Figure 3:**
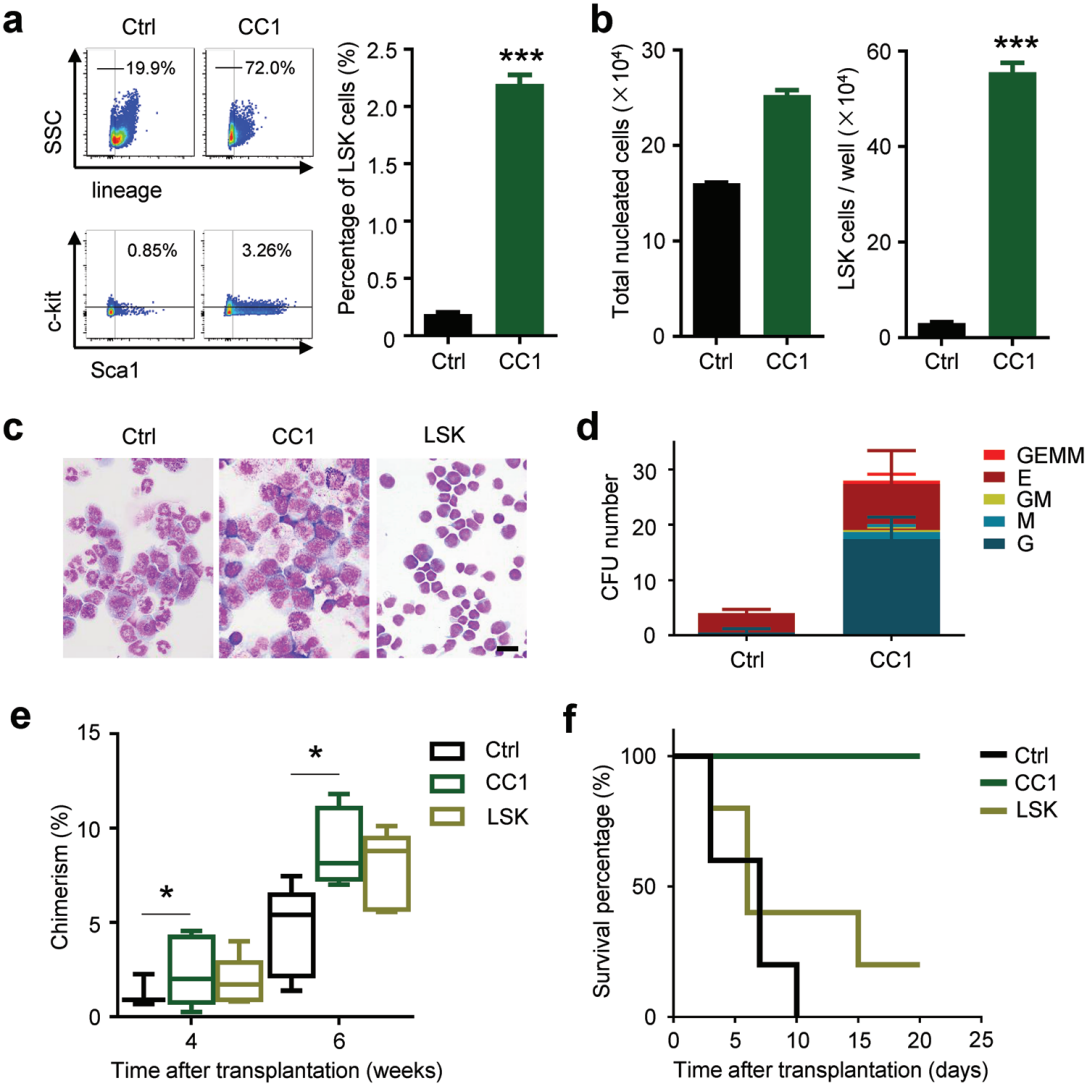
HSPC expansion ex vivo by the chemical cocktail. a) Primary LSK cells isolated from bone marrow were treated with CC1 for 7 d and analyzed for LSK markers. FACS analysis (left). Quantification of the left FACS data (right). ***, *p* < 0.001. b) Total nucleated cell number (left) and absolute LSK cell number (right) after CC1 treatment were quantified (from (a)). ***, *p* < 0.001. c) Giemsa staining of LSK cells treated with CC1 for 7 d, including control one and primary one. Scale bar, 10 µm. d) Expanded HSPCs by CC1 were being with enhanced ability of colony formation. e) LSK cells derived from CD45.2 transgenic mice were treated with CC1 for 7 d ex vivo then were transplanted into CD45.1 mouse. 4 and 6 weeks after transplantation, CD45.2^+^ hematopoietic cells from peripheral blood were monitored by FACS analysis. *, *p* < 0.05. f) Survival of lethally irradiated recipient mice receiving primary LSK cells, LSK cells treated with or without CC1 (7 d) is shown.

Next, we wondered about whether the chemical compounds inducing hematopoietic cell reprogramming could also promote HSPC expansion. Comparing with control group, CC1 significantly enhanced LSK cell expansion ex vivo (**Figure** **3**a). LSK cell number was expanded about 12 times higher in CC1 than that in control (Figure 3b). Comparatively, expanding capacity of CC2 with only 2–3 times more than that of control was relatively lower, which was mainly due to inhibited cell growth (Figure S3a,b, Supporting Information), similar to that in hematopoietic cell reprogramming. Expanded LSK cells from CC1 with 7 d treatment still acquired HSPC phenotype, which is comparable to primary LSK cells (Figure 3c), and robust colony forming ability to CFU‐granulocyte, erythroid, macrophage, and megakaryocyte (CFU‐GEMM), CFU‐E, CFU‐GM, CFU‐M, and CFU‐G (Figure 3d). Previous reports have showed that VPA alone could promote HSPC expansion.^23^ To check the key compound and its optimal combination in CC1, HSPC expansion efficiency was compared among each compound and random combination of component in the chemical cocktail. The results demonstrated that VPA alone or combing with CHIR99021 or Repsox could also significantly enhance LSK cell expansion (Figure S3c, Supporting Information). However, the expanding efficiency of all these three treatments was relatively lower than that of CC1 (Figure S3d,e, Supporting Information). This was also verified by Giemsa staining of expanded LSK cells with diverse chemical treatments (Figure S3f, Supporting Information).

Importantly, to determine whether CC1 treatment could enhance the engraftment of HSPCs, LSK cells from CD45.2 mice treated with or without CC1 for 7 d were injected into sub‐lethally irradiated CD45.1 mice and the reconstitution of CD45.2 CD45^+^ cell frequency in peripheral blood of recipient mice was quantified. 4 and 6 weeks after transplantation, we found that the CC1 treated LSK showed significantly increased engraftment rate, similar to that of noncultured primary LSK cells, comparing with control cells (Figure 3e). Lethally irradiated mice that transplanted with LSK cells cultured with CC1 for 7 d had improved survival compared with mice that received cells cultured with cytokines only or primary LSK cells (Figure 2f).

### 2.5 Single Cell Track Distinguishes Chemical Cocktail‐Induced Hematopoietic Cell Reprogramming from HSPC Expansion

**Figure 4:**
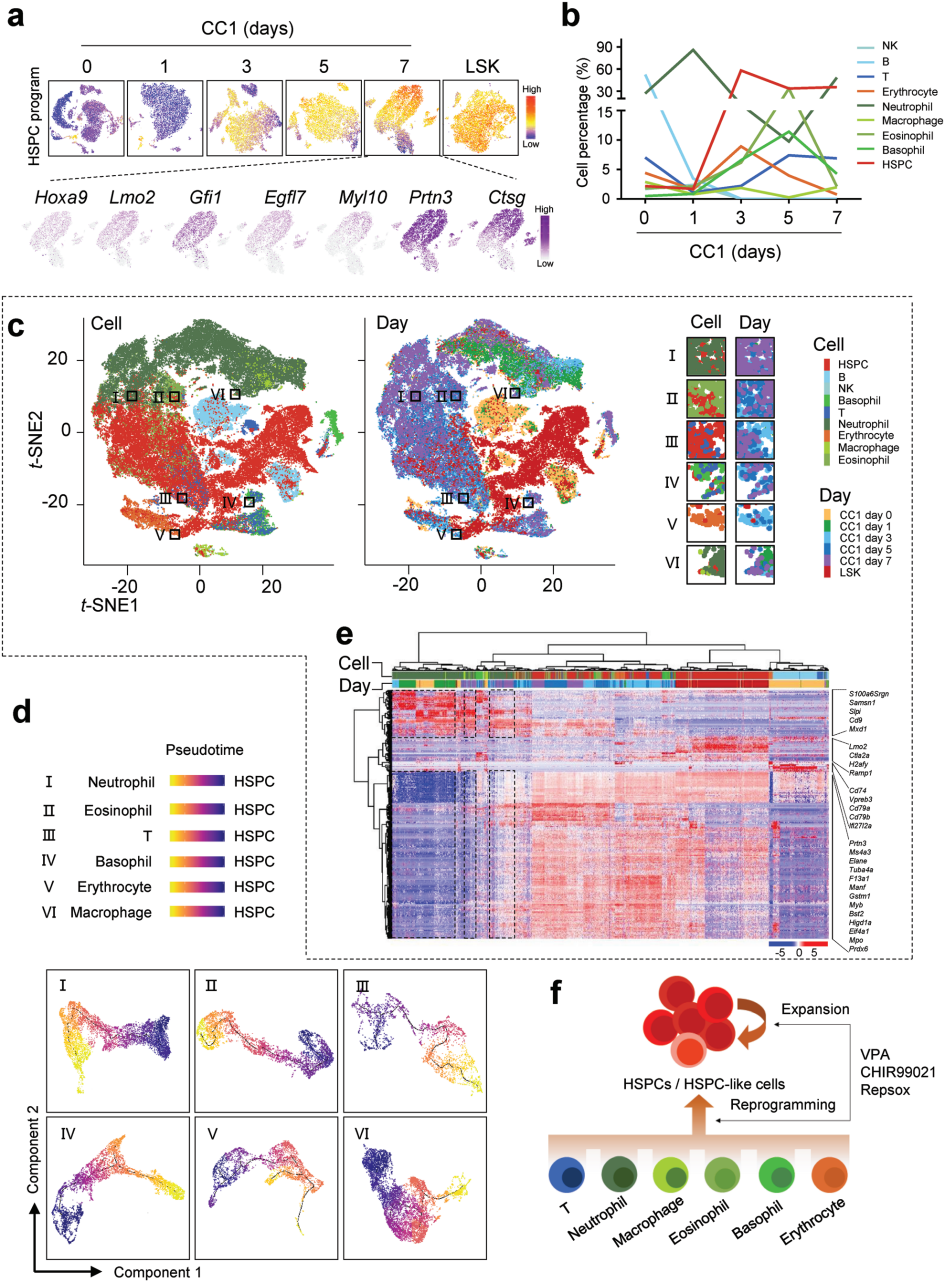
Single cell RNA sequencing of the chemical cocktail induced hematopoietic cell reprogramming and HSPC expansion. a) *t*‐SNE visualization of HSPC signature and gene expression from different time point. The top panel showed HSPC signature of different time points and the control sample. “High” indicate higher mean expression levels using HSPC gene set. The bottom panel showed expression levels of several HSPC signature related genes on day 7. b) Percentage of different cell types in each time point. c) *t*‐SNE visualization of 78302 cells in all time point and control samples, colored by cell types and time point separately. d) UMAP visualization of major trajectories of individual cell types. Charts are colored by pseudotime and edges in the principal graphs that define trajectories are shown as light black line segments. e) Unsupervised clustering heatmap of cells from all cell types and time points. Cell number was unbiased downsampling to 20 000. Euclidean distance was calculated between observation and the agglomeration method ward.D2 was used for hierarchical clustering. f) Schematic model of double effects of the chemical cocktail on hematopoietic cell reprogramming and HSPC expansion.

We next analyzed the hematopoietic cell reprogramming trajectories and HSPC expanding processes by single cell RNA sequencing with 10 × Genomics. Mononuclear cells isolated from wild type mouse bone marrow were treated with CC1. 82 339 individual cells at five time points after chemical treatment, including day 0, 1, 3, 5, and 7, were obtained, with 1935 median genes and 24 719 mean confidently mapped reads per cell (Figure S4a, Supporting Information). Based on *t*‐distributed stochastic neighbor embedding (*t*‐SNE) plot analysis, nine transcriptionally distinct clusters of cell lineages were identified in day 0 starting hematopoietic cells:^24^, ^25^ HSPCs, T cells, B cells, NK cells, erythrocytes, macrophages, basophils, eosinophils, and neutrophils (Figure S4b, Supporting Information). On day 0, HSPCs only accounted for 2.17% of the whole cell population. Following CC1 treatment, cells acquiring HSPC program constituted the major cell type in the whole population from day 3 to 7 (**Figure** **4**a), with myeloid cells remaining as the second cell type, B cells and NK cells almost disappearing (Figure S4b, Supporting Information; Figure 4b). Clustering analyses at diverse time points also demonstrated that HSPC genes, such as *Hoxa9*, *Lmo2*, *Gfi1*, *Egfl7*, *Myl10*, *Prtn3*, and *Ctsg*, were successively activated in major cells from day 3.

In *t*‐SNE projection of whole cells on diverse days, it demonstrated that cell reprogramming process might happen to six different hematopoietic starting cells (Figure 4c). To further dissect the cell reprogramming toward HSPCs, we analyzed cells using Monocle 2/3 to reconstruct the trajectory in a pseudo temporal manner. It was found that lineages of neutrophils, eosinophils, basophils, macrophages, erythrocytes, and T cells have the capacity to progress toward HSPCs under our experimental conditions (Figure 4d). These findings were supported by hierarchical clustering calculated with primary LSK cells, which demonstrated that expression of initiating cell specific genes was gradually and partially inactivated in the cells with activated HSPC genes from day 3 to day 7 after CC1 treatment (Figure 4e). We define these induced cells with HSPC program, which shared a highly similar global expression profile with primary LSK cells (Figure S4c, Supporting Information), as HSPC‐like cells. On day 7, 84.73% eosinophils, 70.81% erythrocytes, 41.05% T cells, 28.97% neutrophils, 18.16% basophils, and 4.74% macrophages acquired HSPC gene expression profile (Figure S4d, Supporting Information). Neutrophil‐, T‐, erythrocyte‐, basophil‐, macrophage‐, and eosinophil‐derived HSPC‐like cells account for 24.49%, 4.93%, 0.92%, 1.35%, and 3.14% of the total cells being with HSPC program individually, with expanded HSPCs accounting for 65.01%. Thus, beyond hematopoietic cell reprogramming, major HSPCs came from original HSPC expansion in CC1 treated bone marrow mononuclear cells (Figure S4e, Supporting Information).

### 2.6 Different Hematopoietic Cell Lineages Acquire Diverse Reprogramming Potential

**Figure 5:**
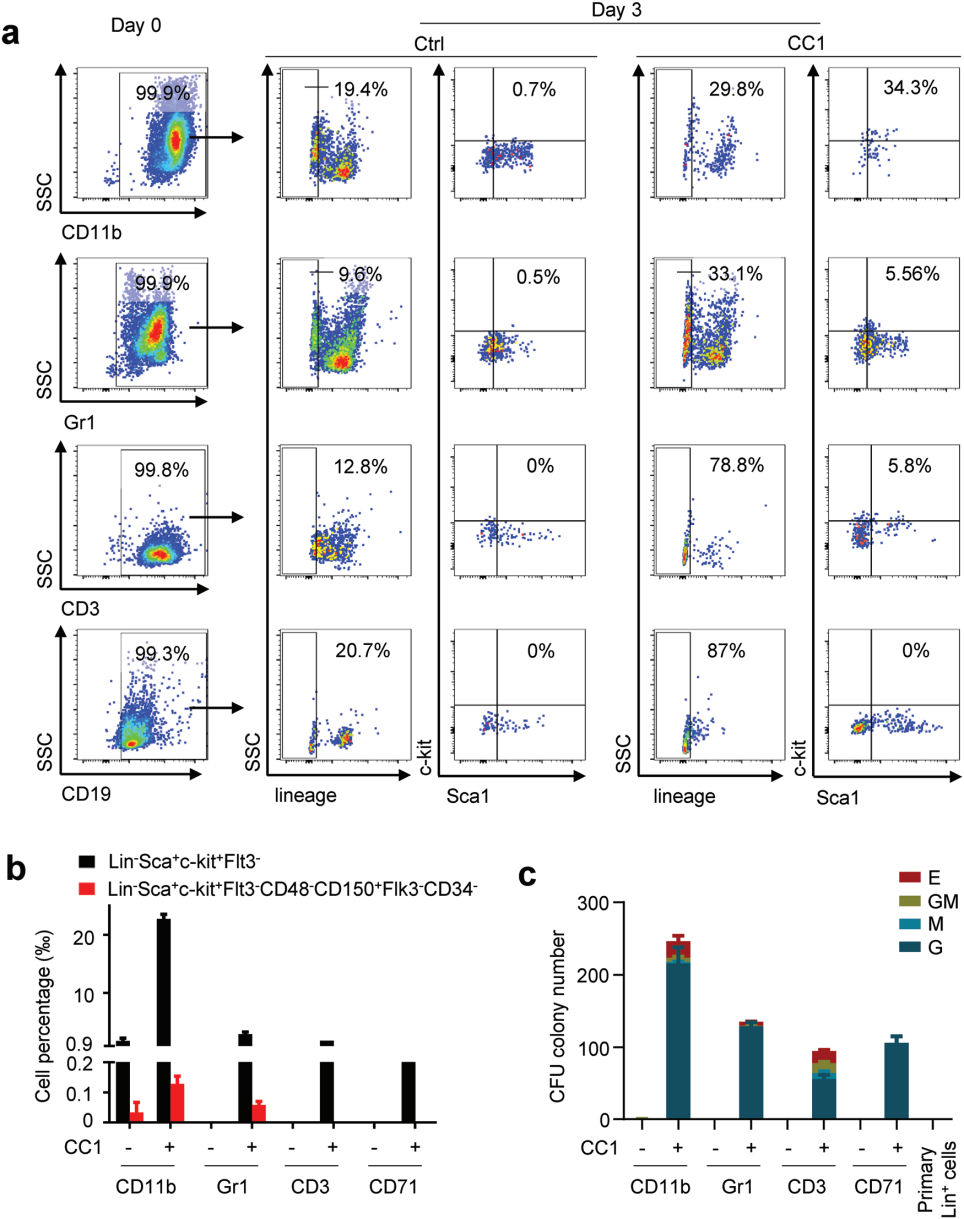
Reprogramming potential of diverse hematopoietic cell lineages. a) CD11b^+^ macrophages, Gr1^+^ neutrophils, CD19^+^ B cells, and CD3^+^ T cells isolated from bone marrow were treated with CC1 individually for 3 d then were analyzed by FACS for LSK markers. Data for CD71^+^ erythrocytes were not shown here due to that no live cells was observed in ctrl on day 3. b) Detection of long‐term LSK cells from CD11b^+^, Gr1^+^, CD19^+^, and CD71^+^ hematopoietic cells treated with or without CC1 for 10 d. c) Reprogrammed‐individual hematopoietic cells were cultured in M3434 for 14 d to investigate the colony formation ability.

Next, we sought to determine the reprogramming potential of individual hematopoietic cell lineages being converted back into HSPC‐like cells by the chemical cocktail. We isolated CD11b^+^ macrophages, Gr1^+^ neutrophils, CD19^+^ B cells, CD3^+^ T cells, and CD71^+^ erythrocytes respectively from bone marrow‐derived hematopoietic cells via double‐time cell sorting to eliminate any LSK cell contamination. These purified cells were treated with CC1 in cytokines. 3 d after treatment, the percentage of lineage negative cells usually representing stem/progenitor cell property in hematopoietic cells was higher in CC1 than that in control for all these five types of cells (**Figure** **5**a). For the lymphoid cells here, LSK cells were only detected in CC1 treated T cells, but not B cells. Comparatively, LSK cells were induced in both myeloid cells here by CC1 on day 3 and day 7 (Figure S5a, Supporting Information). Among these myeloid‐derived induced LSK cells, long‐term hematopoietic stem cells phenotypically characterized as Lin^−^Sca^+^c‐kit^+^Flt3^−^CD48^−^CD150^+^Flk3^−^CD34^−^ were also detected (Figure 5b; Figure S5b, Supporting Information). Colony formation assay showed that both CD11b‐ and CD3‐derived LSK cells acquired the ability to form CFU‐E, CFU‐GM, CFU‐M, and CFU‐G, while Gr1‐derived LSK cells formed CFU‐E and CFU‐G, and CD71‐derived cells only form CFU‐G (Figure 5c; Figure S5c, Supporting Information).

Together, these data demonstrate that different differentiated hematopoietic cells acquire diverse potential to be reprogrammed back into cells with progenitor cell property by the chemical cocktail.

## 3 Discussion

In this study, combination of chemical compounds activating Sox2 expression successfully reprograms mouse fibroblasts into hematopoietic cells, which is through hemogenic stage. This is consistent with previous reports that both pluripotent stem cell differentiation and somatic cell reprogramming into hematopoietic cells mediated by overexpression of transcription factors require hemogenic cell generation.^4^, ^26^ Endothelial‐to‐hematopoietic transition observed during hematopoietic development in vivo is also considered as required procession.^6^, ^27^ Among those reports, cell reprogramming from endothelium to HSPCs is more efficient than that from fibroblasts. Not like these HSPCs induced from endothelium or fibroblasts, induced HSPCs from pre/pro‐B cells even function better. All these data suggest that cell reprogramming within the same developmental lineage often generate better functional cells efficiently than that between trans‐lineages. It explains that why HSPC‐like cells with multihematopoietic differentiation potential were generated from myeloid cells or lymphoid cells, but only hemogenic cells with limited developmental potential were induced from fibroblasts, by the same chemical compound cocktail here.

This cocktail was initially discovered to induce neural stem or progenitor cell generation,^11^, ^12^ which belongs to ectoderm. It is developmentally different direction from hematopoietic cell generation, which belongs to mesoderm. Same inducer with diverse induced cells might be due to exogenous factors. Many reports show that microenvironmental factors or culturing conditions play an important role in the choice of direction of cell fate conversion, even with the same transcription factors, such as Sox2 or Oct4.^20^, ^28^, ^29^, ^30^, ^31^ Our previous data also demonstrate that growth factors or cytokines modulate the chemical induced cell reprogramming.^13^ With the cutting‐edge techniques, lineage tracing and single cell analysis, canonical development trajectory is finely revised recently. Ectoderm and mesoderm developmentally come from the same lineage and even mesectoderm‐committed progenitor cells are identified.^32^, ^33^, ^34^ Thus, it is speculated that the chemical cocktail used here might induce somatic cells into progenitor cells, which further develop into ectodermal neural cells or mesodermal hematopoietic cells individually under specific conditions.

HSPC expansion can be promoted by single chemical compounds.^35^, ^36^ However, more and more data show that combination of chemical compounds, which modulate different signaling targets, often shows synergistic effect on HSPC expansion with more efficiency than each one alone.^37^, ^38^, ^39^ In our chemical recipe, VPA as histone deacetylases inhibitor promoting HSPC proliferation is reported before,^23^, ^40^, ^41^ and this effect is further enhanced by addition of GSK3β inhibitor,^37^ which is also verified in our experiment. Beyond cell proliferation, VPA can also induce human CD34^+^CD90^−^ HSPC subclone reprogramming into CD34^+^CD90^+^ HSPC subclone. The latter cell type acquires better capacity of hematopoietic reconstruction.^42^ Vice versa, cocktail of chemical compounds promoting cell reprogramming to pluripotency is shown to maintain human HSPCs, although whether hematopoietic reprogramming is induced in that study is not investigated.^39^ These data and studies collectively demonstrate that combination of chemical compounds with diverse molecular mechanisms might potentially and simultaneously promotes HSPC expansion and reprogramming to HSPC generation.

Taken together, we show for the first‐time that somatic cell reprogramming into hemogenic cells can be induced by chemical compounds without introduction of exogenous transcription factors. We also show that promoting HSPC expansion and inducing differentiated hematopoietic cell reprogramming back into progenitors might be achieved at the same time by chemical cocktails (Figure 4f). Considering the golden standard for HSPC definition still depends on hematopoietic reconstitution in vivo, whether the chemical‐expanded HSPCs acquire long‐term hematopoietic stem cell function, and the chemical‐induced HSPC‐like cells acquire short‐term even long‐term HSPC property in vivo, remaining to be further investigated. Based on completion of all the above assays, it will provide a potentially advanced strategy for generating HSPCs.

## 4 Experimental Section

*Cell Culture*: Mouse embryonic fibroblasts were isolated from E13.5 mouse embryos. Briefly, embryonic tissues without head, limbs, visceral tissues, gonads, or vertebral column were scissored into pieces and trypsinized. Fibroblasts were cultured in Dulbecco's Modified Eagle's Medium (DMEM) supplemented with 10% fetal bovine serum (FBS), 1 × 10^−3^ m GlutaMAX, 0.1 × 10^−3^ m nonessential amino acid, 100 units mL^−1^ penicillin, and 100 mg mL^−1^ streptomycin at 37 °C with 5% CO_2_. All cells at early passages (less than three passages) were used for further experiment.

*Mice*: Mice C57BL/6 and CD45.1 were purchased from Sino‐British SIPPR/BK Lab. Animal Ltd (Shanghai, China). All mice were housed and cared in pathogen‐free condition. All experiments were carried in accordance with the National Institutes of Health Guide for the Care and Use of Laboratory Animals and were approved by the Laboratory Animal Resource Center of Shanghai Jiao Tong University School of Medicine.

*Hematopoietic Cell Reprogramming*: Freshly isolated Scl‐GFP^−^ bone marrow cells were plated in M5300 medium supplemented with murine SCF (50 ng mL^−1^), mFlt3L (50 ng mL^−1^), mIL‐3 (20 ng mL^−1^), mIL‐6 (20 ng mL^−1^), 1 × 10^−6^ m Hydrocortisone and 1% penicillin–streptomycin solution at the density of 5 × 10^5^ mL^−1^ in 96 well plates. Spleen Scl‐GFP^−^ cells were plated at a density of 7 × 10^5^ mL^−1^ in the same procedure described above. Wells were examined manually to pick well with no GFP^+^ cell contamination in 18 h. The cells were then cultured for 7 d in medium supplemented with 500 × 10^−6^ m VPA, 3 × 10^−6^ m CHIR99021, and 1 × 10^−6^ m Repsox at 37 °C in a humidified atmosphere and 5% CO_2_ in air.

*HSPC Expansion*: LSK (lineage^−^, Sca1^+^, c‐kit^+^) cells were sorted by FACS and seeded in M5300 medium supplemented with murine SCF (50 ng mL^−1^), mFlt3L (50 ng mL^−1^), mIL‐3 (20 ng mL^−1^) and mIL‐6 (20 ng mL^−1^), 1 × 10^−6^ m hydrocortisone and 1% penicillin–streptomycin solution or Iscove's Modified Dulbecco's Medium (IMDM) with 10% FBS, mSCF (50 ng mL^−1^), mFLT3L (50 ng mL^−1^), mIL‐3 (20 ng mL^−1^), mIL (20 ng mL^−1^), 100 units mL^−1^ penicillin, and 100 mg mL^−1^ streptomycin at the density of 2 × 10^4^ mL^−1^. Cells were cultured for 7 d in medium supplemented with 500 × 10^−6^ m VPA, 3 × 10^−6^ m CHIR99021, and 1 × 10^−6^ m Repsox at 37 °C in a humidified atmosphere and 5% CO_2_ in air. Medium was changed every other day.

*Colony Formation Assay*: For HSPC expansion assay, total nucleated cells were harvested and counted on Countstar. Cells, seeded at a density of 1.5 × 10^4^ mL^−1^, were transferred into 12‐well culture plate in 1 mL M3434 methylcellulose (Stem cell Technologies) supplemented with 100 units mL^−1^ penicillin and 100 mg mL^−1^ streptomycin. For hematopoietic cell reprogramming experiments using GFP negative bone marrow cells, total nucleated cells were collected and GFP positive cells were sorted by FACS upon 7 d of culture. 2 × 10^4^ cells were seeded in one well in 12‐well plate in 1 mL M3434 methylcellulose as described above. Colonies were identified and counted on Day 8 to Day 10.

*Giemsa Staining*: Total nucleated cells were harvested and counted on Countstar. Resuspend cell preparation to a density of 7.5 × 10^5^ mL^−1^ and 200 ul of each cell suspension was used to spin in Cytocentrifuge (Shandon) (800 rpm, 5 min). Slides were allowed air dry before staining. Slides were first stained in undiluted Giemsa stain for 1 min followed by a 1:1 mixture of Giemsa stain and Phosphate Buffer for 4 min. Slides were rinsed in water thoroughly and allowed air dry.

*Flow Cytometry and Cell Sorting*: Multicolor analysis of HSPCs were performed on BD LSKFortessa X‐20. Cells were collected and stained with APC‐CD117 (BD), PE‐Sca1 (BD), BV421 mouse lineage cocktail (BD), BV711‐c‐kit (Biolegend), PE‐Sca1 (BD), or BV421‐mouse lineage cocktail (BD) in PBS at 4 °C for 30 min. Cells were washed with PBS and stained with 7AAD before detection. Results were analyzed by Flowjo_V10 software.

8‐week male C57 mice were killed by cervical dislocation. Bone marrow cells were flushed out from long bones (femora and tibia). For HSPC sorting, cells were stained with BV421 lineage cocktail (BD), APC‐CD117 (BD), and PE‐Sca1 (BD) for 30 min after erythrocyte lysis. To sort lineage^+^ cells, bone marrow cells were stained with Alexa700‐Gr1 (Biolegend), APC‐CD11b (Biolegend), BV421‐CD3 (Biolegend), or BV510‐CD19 (Biolegend) with the same procedure described above. For GFP negative cell sorting, no antibody staining is needed. Cells were washed by PBS and forced through 100 µm cell strainer to remove any clots before sorting. Cells were sorted twice by FACSAria lll (BD) to yield higher purity. Sorted CD11b^+^ cells, Gr1^+^ cells were analyzed by flow cytometry to test cell purity and LSK cell contamination.

*Quantitative Real‐Time PCR*: Total RNAs were extracted from cells using Trizol reagent (Sigma‐Aldrich) according to the manufacture's protocols. To obtain cDNA, RNA was reverse‐transcribed by M‐MLV reverse transcriptase and random hexamers. cDNA, 2 × 3 PCR Mix and Eva Green were mixed and analyzed with MX3000P Stratagene PCR machine. The relative mRNA expression values were normalized against the inner control (GAPDH).

*HSPC Transplantation In Vivo*: All mice used in transplantation experiment are 8–10 weeks of age and C57BL/6 background. Recipient CD45.1 mice were treated with 375 cGy radiation for 64 s twice with a break for at least 3 h. Nucleated cells derived from 1000 starting LSK cells were stained with 7AAD and 7AAD^−^ cells were kept for tail vein injection. Sorted CD45.2 7AAD^−^ cells were transplanted along with 2 × 10^5^ CD45.1 marrow competitor. Percentage of CD45.2 cells in peripheral blood of recipient mice was monitored 4/6 weeks after transplantation.

*RNA Sequence and Analysis*: Total RNA was extracted from ≈1 million cells. 3 mg of RNA per sample was used as input sequencing library preparation using NEBNext Ultra RNA Library Prep Kit for Illumina following manufacturer's recommendations. Multiplexed libraries were mixed and sequenced by Illumina Hiseq 4000 at the depth of 50–60 million 150 bp pair‐end reads. The output transcript compatibility counts were used for Gene‐based clustering.

*Quantification and Statistics*: All experiments were repeated at least three times. All quantified data were statistically analyzed and presented as mean ± SEM. Unless otherwise stated, one‐way ANOVA was used to calculate statistical significance with *p*‐values are detailed in figure legends.

*Single Cell RNA Sequence and Data Analysis*: Viable cells from culture or bone marrow isolation were collected and analyzed on the Chromium system (10 × Genomics) according to the manufacturer's instructions for an expected capture rate of ≈15 000 single cells per sample.

Raw reads of single‐cell sequencing in FASTQ format (text‐based sequencing data file format that stores both raw sequence data and quality scores) were aligned to mouse reference (mm10, v3.0.0) using Cell Ranger software (v3.0.2), which is provided by 10 × Genomics (https://support.10xgenomics.com). The same software was used for barcode assignment and unique molecular identifier counting using the parameter—expect cells 10 000. R‐package Seurat was used to read raw count data into a Seurat object and performed analyzing workflow.^43^ Cells expressed less than 300 genes or over 0.2 mitochondrial RNA percentage were filtered out as these might represent doublets or cell debris. The top 2000 genes with the highest variance were selected and the digital gene‐expression matrix was renormalized after gene filtering. These genes were also enrolled in the calculation of principal component analysis (PCA). The principal components with *p*‐value less than 0.01 were used in a graph‐based clustering algorithm based on the built‐in jackstraw analysis. Resolution to topological divide cells into different clusters was set to 1.5. For visualization purpose, two methods were performed to reduce dimensions for single‐cell expression profile: *t*‐SNE and Uniform Manifold Approximation and Projection (UMAP). Cell types were finally identified by significantly expressed markers of each cluster with (p_val_adj) ≤ 0.05 and average log fold change (avg_logFC) ≥ 0.5. After all major cell types were identified, single‐cell sequencing data from different time point were merged together using IntegrateData function in Seurat. Batch effects were corrected using the built‐in modules of Seurat package.

Monocle 2 and Monocle 3 were used to reconstruct differentiation trajectory.^44^ An aggregated gene‐expression matrix was constructed based on Seurat results and differentially expressed genes (DEGs) across different time points were identified using Monocle 2. Genes with *q* value less than 0.05 were used to construct the pseudotime trajectory using UMAP method.

## Conflict of Interest

The authors declare no conflict of interest.

## Supporting information

Supporting Information
