# Supplementary material for: Chemical Cocktail Induces Hematopoietic Reprogramming and Expands Hematopoietic Stem/Progenitor Cells
Source: Adv Sci (Weinh). 2019 Nov 11;7(1):1901785. doi: 10.1002/advs.201901785 (PMC6947705; doi:10.1002/advs.201901785)
Supplement: Supplementary file 1 — Supporting Information [file ADVS-7-1901785-s001.pdf]

Copyright WILEY-VCH Verlag GmbH & Co. KGaA, 69469 Weinheim, Germany, 2019.

ADVANCED SCIENCE
Open Access

Supporting Information

for *Adv. Sci.,* DOI: 10.1002/advs.201901785

Chemical Cocktail Induces Hematopoietic Reprogramming and Expands Hematopoietic Stem/Progenitor Cells

Yi Zhou, Xingli Zhu, Yuting Dai, Shumin Xiong, Chuijin Wei, Pei Yu, Yuewen Tang, Liang Wu, Jianfeng Li, Dan Liu, Yanlin Wang, Zhu Chen, Sai-Juan Chen, Jinyan Huang,* and Lin Cheng**

Supporting Information

Figure legends

Figure S1. Chemical Activation of Scl-GFP in Mouse Fibroblasts. a) qRT-PCR analysis of expression of *Sox2* and *Cd34* in mouse fibroblasts after chemical cocktail treatment for different days. b) Depletion of Sci-GFP$^+$ cells and CD45$^+$ cells in fibroblasts. Representative figures (up). FACS analysis (down). Scale bar, 50 μm. c) Schematic model of chemical induction of mouse embryonic fibroblasts isolated from *Scl*-GFP transgenic mouse. d) Heatmap of hemogenic gene expression and fibroblast gene expression from RNA sequence data. Red indicates increased expression and green indicates decreased expression comparing with that in ctrl. e) FACS analysis of stem/progenitor related markers in chemical cocktail induced Scl-GFP$^+$ cells. f) FACS analysis of Scl-GFP$^+$ cells 5 days after Scl-GFP$^-$/CD45$^-$ fibroblasts cultured with or without supporting cells or coating matrix, and with diverse chemical cocktail treatments. g) Quantification of data from f). *, $P<0.05$. **, $P<0.01$. h) Chemical cocktail induced Scl-GFP$^+$ cells on E4EC differentiated into CD45$^+$CD11b$^+$ cells, analyzing by FACS.

Figure S2. Chemical Activation of HSPC Program in Differentiated Hematopoietic Cells. a) Schematic model of isolating Scl-GFP$^-$ hematopoietic cells from bone marrow or spleen. b) Representative figures of bone marrow derived Scl-GFP$^-$ mononuclear cells treated with CC1 or CC2 for 7 days. c) Representative figures

of spleen derived Scl-GFP$^-$ mononuclear cells treated with CC1 or CC2 for 7 days. Scale bar, 50 μm. d) Spleen derived Scl-GFP$^-$ hematopoietic cells were reprogrammed into Scl-GFP$^+$ cells by the treatment of chemical cocktails on day 7. Representative data of FACS analysis (left). Quantification of percentage of Scl-GFP$^+$ cells (middle). Quantification of Scl-GFP$^+$ cell number (right). e) Detection of LSK cell numbers in chemical induced Scl-GFP$^+$ cells from spleen derived Scl-GFP$^-$ hematopoietic cells. f) Gene ontology analysis of the diverse gene clusters in figure 2d.

Figure S3. Chemical Expansion of HSPCs. a) FACS analysis of LSK cell percentage 7 days after LSK cells treated with CC2. ***, $P<0.001$. b) Total nucleated cell number was counted (left) and LSK cell number was quantified (right) 7 days after HSPCs treated with CC2. *, $P<0.05$. **, $P<0.01$. c) FACS analysis of LSK cells, 7 days after primary LSK cells treated with single component or combination of each component among CC1. d) Quantification of data from (c). e) Total nucleated cell number was counted (left) and LSK cell number was quantified in (c). f) Representative figures for Giemsa staining of cells, 7 days after primary LSK cells treated with single component or combination of each component among CC1. Scale bar, 10 μm.

Figure S4. Single Cell Analysis of Chemical Cocktail Treated Hematopoietic Cells. a) Distribution of the number of confidently mapped reads detected per cell of all time point and the control sample. The top panel showed the distribution of genes, and the second panel showed the distribution of UMIs, whereas the third panel showed the

scatter plots between the number of genes and UMIs in each cell from all time point. b) *t*-SNE visualization of major cell types in each time point and control sample. c) Scatter plots of the global gene expression profile between terminal cells on the successful reprogramming trajectory branch of major cell types and primary LSK cells. d) Cell reprogramming efficiency of each hematopoietic cells was calculated by comparing each type of hematopoietic cell-derived HSPC-like cell number with its total counterpart on day 7. e) Percentage of induced HSPC-like cells and expanded HSPCs in total cells with HSPC program on day 7.

Figure S5. Morphology and Functional Characterization of Reprogrammed Hematopoietic Cells. a) $CD11b^+$ macrophages and $Gr1^+$ neutrophils isolated from bone marrow were treated with CC1 individually for 7 days then were analyzed by FACS for LSK markers. b) Representative figures for Giemsa staining of primary cells and the cells being treated with or without CC1 for 7 days. Scale bar, 10 μm. c) Representative figures for the colony formation of reprogrammed-individual hematopoietic cells.

Figure S1

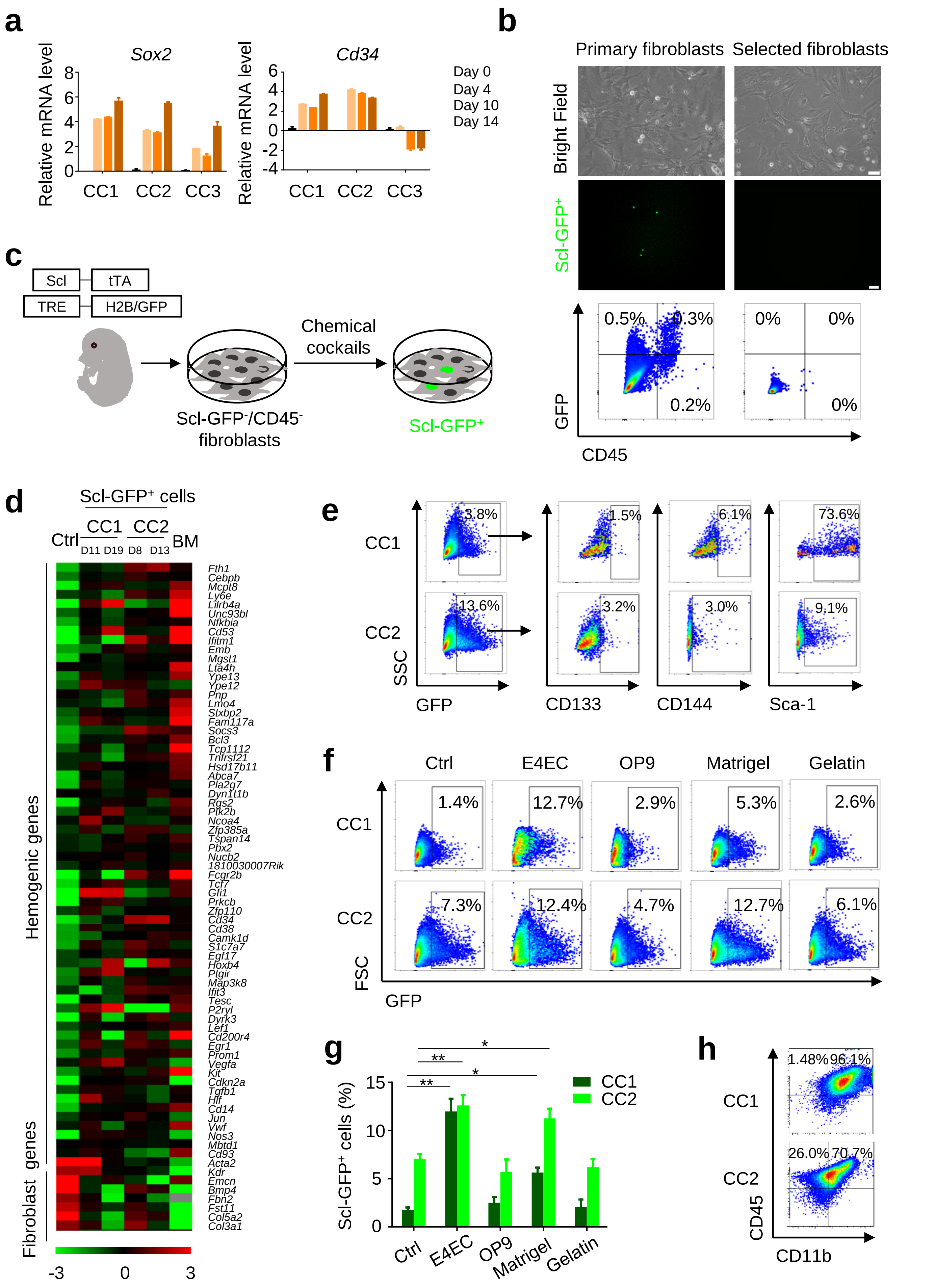
a
Sox2
Cd34
Relative mRNA level
CC1 CC2 CC3
Day 0
Day 4
Day 10
Day 14
b
Primary fibroblasts
Selected fibroblasts
Bright Field
Scl-GFP+
0.5%
0.3%
0.2%
0%
GFP
CD45
c
Scl
tTA
TRE
H2B/GFP
Chemical cockails
Scl-GFP-/CD45- fibroblasts
Scl-GFP+
d
Scl-GFP+ cells
Ctrl
CC1
CC2
D11 D19 D8 D13
BM
Hemogenic genes
Fibroblast genes
Fth1 Cebpb Mcpt8 Ly6e Lilrb4a Unc93bl Nfkbia Cd53 Ifitm1 Emb Mgst1 Lta4h Ype13 Ype12 Pnp Lmo4 Stxbp2 Fam117a Socs3 Bcl3 Tcp1112 Tnfrsf21 Hsd17b11 Abca7 Pla2g7 Dyn1t1b Rgs2 Ptk2b Ncoa4 Zfp385a Tspan14 Pbx2 Nucb2 1810030007Rik Fcgr2b Tcf7 Gfi1 Prkcb Zfp110 Cd34 Cd38 Camk1d Slc7a7 Egfl7 Hoxb4 Ptgir Map3k8 Ifit3 Tesc P2ryl Dyrk3 Lef1 Cd200r4 Egr1 Prom1 Vegfa Kit Cdkn2a Tgfb1 Hlf Cd14 Jun Vwf Nos3 Mbtd1 Cd93 Acta2 Kdr Emcn Bmp4 Fbn2 Fst11 Col5a2 Col3a1
-3 0 3
e
CC1
CC2
3.8%
1.5%
6.1%
73.6%
13.6%
3.2%
3.0%
9.1%
SSC
GFP
CD133
CD144
Sca-1
f
Ctrl E4EC OP9 Matrigel Gelatin
CC1
CC2
1.4% 12.7% 2.9% 5.3% 2.6%
7.3% 12.4% 4.7% 12.7% 6.1%
FSC
GFP
g
Scl-GFP+ cells (%)
CC1
CC2
Ctrl E4EC OP9 Matrigel Gelatin
h
CC1
CC2
1.48% 96.1%
26.0% 70.7%
CD45
CD11b

Figure S2

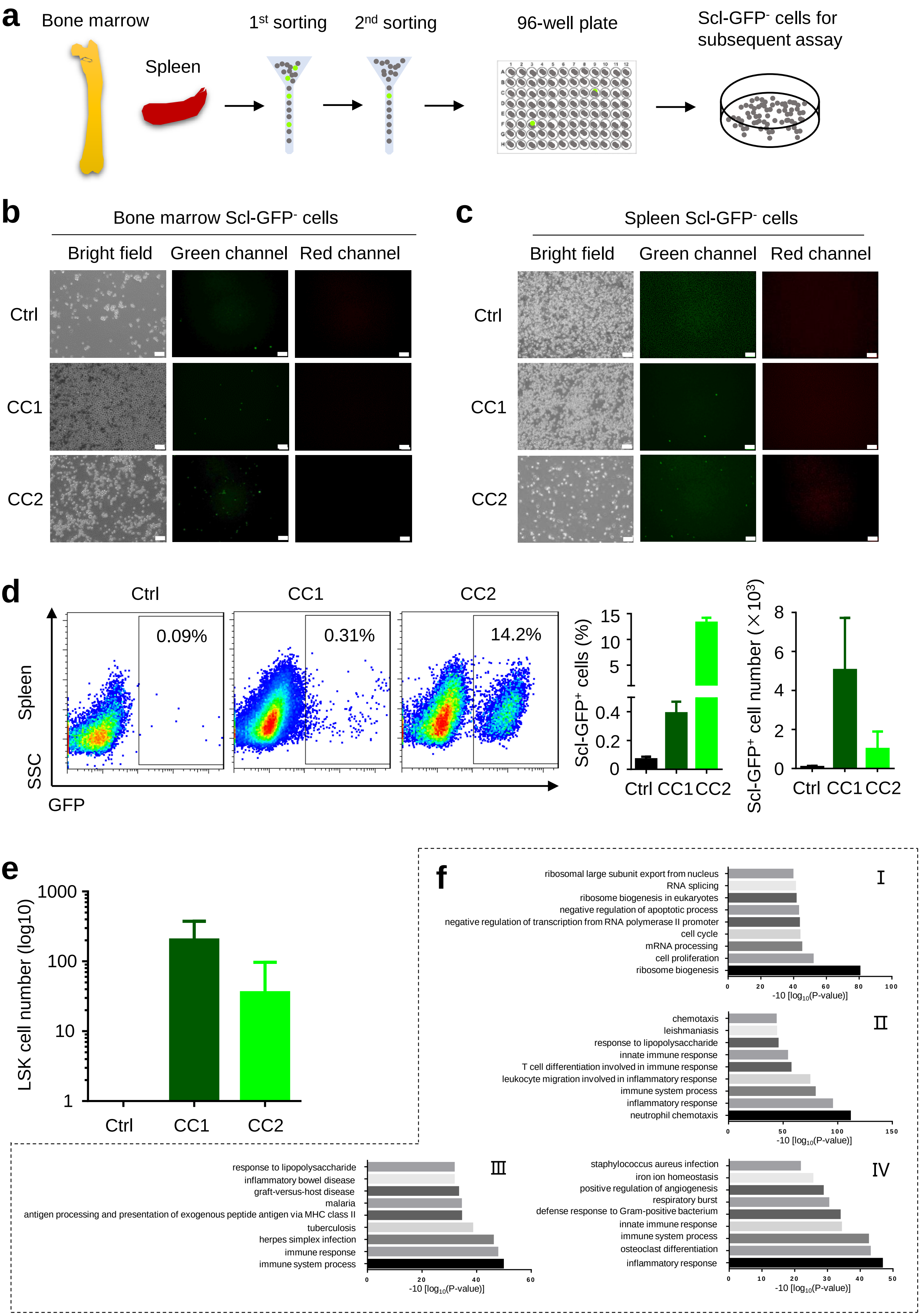
a
Bone marrow
Spleen
1st sorting
2nd sorting
96-well plate
Scl-GFP- cells for subsequent assay
b
Bone marrow Scl-GFP- cells
Bright field
Green channel
Red channel
Ctrl
CC1
CC2
c
Spleen Scl-GFP- cells
Bright field
Green channel
Red channel
Ctrl
CC1
CC2
d
Ctrl
CC1
CC2
0.09%
0.31%
14.2%
Spleen
SSC
GFP
Scl-GFP+ cells (%)
Scl-GFP+ cell number (×10³)
Ctrl CC1 CC2
e
LSK cell number (log10)
1000
100
10
1
Ctrl
CC1
CC2
f
I
ribosomal large subunit export from nucleus
RNA splicing
ribosome biogenesis in eukaryotes
negative regulation of apoptotic process
negative regulation of transcription from RNA polymerase II promoter
cell cycle
mRNA processing
cell proliferation
ribosome biogenesis
-10 [log10(P-value)]
II
chemotaxis
leishmaniasis
response to lipopolysaccharide
innate immune response
T cell differentiation involved in immune response
leukocyte migration involved in inflammatory response
immune system process
inflammatory response
neutrophil chemotaxis
-10 [log10(P-value)]
III
response to lipopolysaccharide
inflammatory bowel disease
graft-versus-host disease
malaria
antigen processing and presentation of exogenous peptide antigen via MHC class II
tuberculosis
herpes simplex infection
immune response
immune system process
-10 [log10(P-value)]
IV
staphylococcus aureus infection
iron ion homeostasis
positive regulation of angiogenesis
respiratory burst
defense response to Gram-positive bacterium
innate immune response
immune system process
osteoclast differentiation
inflammatory response
-10 [log10(P-value)]

Figure S3

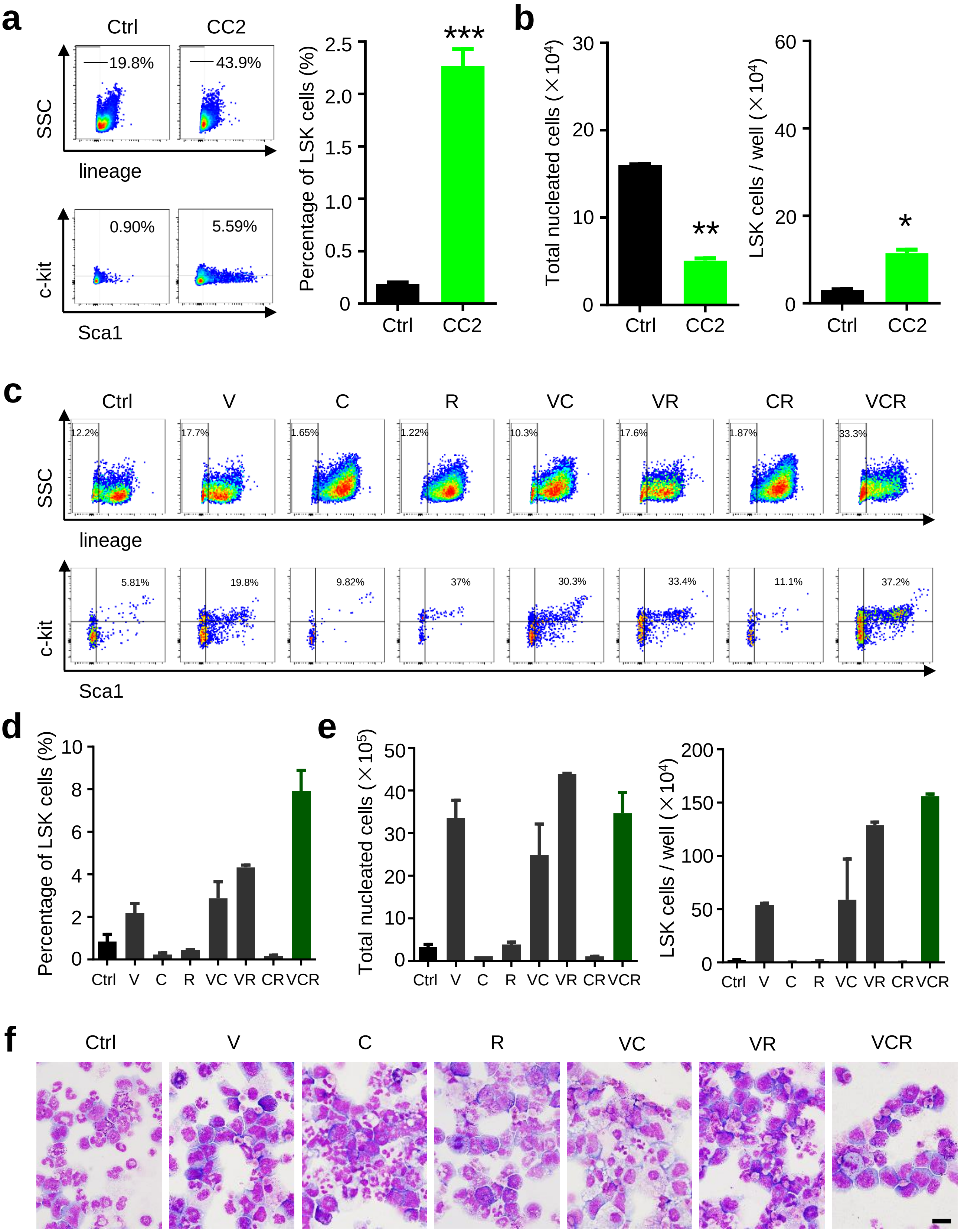

a
Ctrl
CC2
19.8%
43.9%
SSC
lineage
0.90%
5.59%
c-kit
Sca1
Percentage of LSK cells (%)
***
b
Total nucleated cells (×10^4)
**
LSK cells / well (×10^4)
*
c
Ctrl V C R VC VR CR VCR
12.2%
17.7%
1.65%
1.22%
10.3%
17.6%
1.87%
33.3%
5.81%
19.8%
9.82%
37%
30.3%
33.4%
11.1%
37.2%
d
e
Total nucleated cells (×10^5)
f

Figure S4

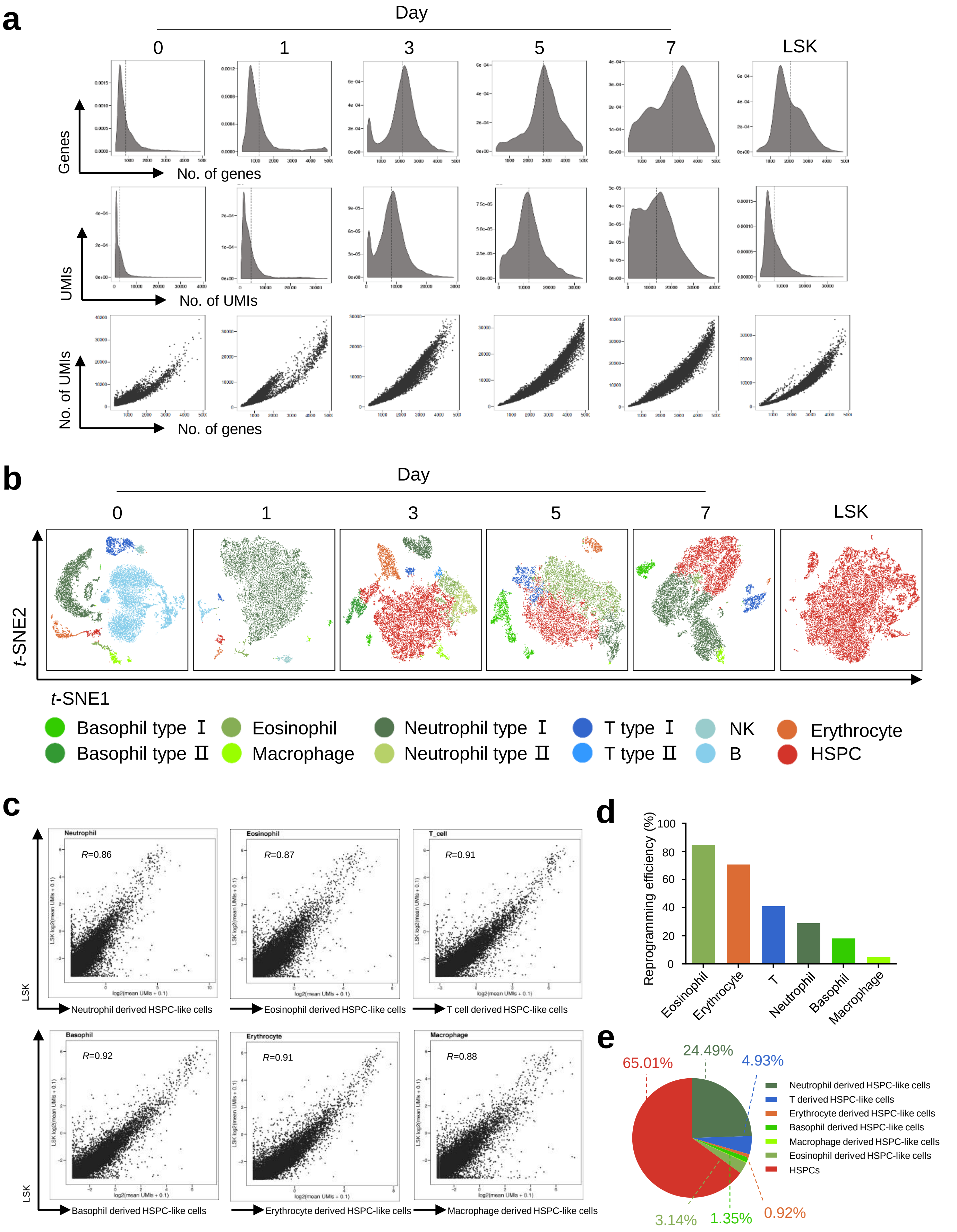
a
Day
0
1
3
5
7
LSK
Genes
No. of genes
UMIs
No. of UMIs
No. of UMIs
No. of genes
b
Day
0
1
3
5
7
LSK
t-SNE2
t-SNE1
Basophil type Ⅰ
Basophil type Ⅱ
Eosinophil
Macrophage
Neutrophil type Ⅰ
Neutrophil type Ⅱ
T type Ⅰ
T type Ⅱ
NK
B
Erythrocyte
HSPC
c
Neutrophil
R=0.86
Eosinophil
R=0.87
T_cell
R=0.91
Basophil
R=0.92
Erythrocyte
R=0.91
Macrophage
R=0.88
LSK
Neutrophil derived HSPC-like cells
Eosinophil derived HSPC-like cells
T cell derived HSPC-like cells
Basophil derived HSPC-like cells
Erythrocyte derived HSPC-like cells
Macrophage derived HSPC-like cells
d
Reprogramming efficiency (%)
100
80
60
40
20
0
Eosinophil
Erythrocyte
T
Neutrophil
Basophil
Macrophage
e
65.01%
24.49%
4.93%
3.14%
1.35%
0.92%
Neutrophil derived HSPC-like cells
T derived HSPC-like cells
Erythrocyte derived HSPC-like cells
Basophil derived HSPC-like cells
Macrophage derived HSPC-like cells
Eosinophil derived HSPC-like cells
HSPCs

Figure S5

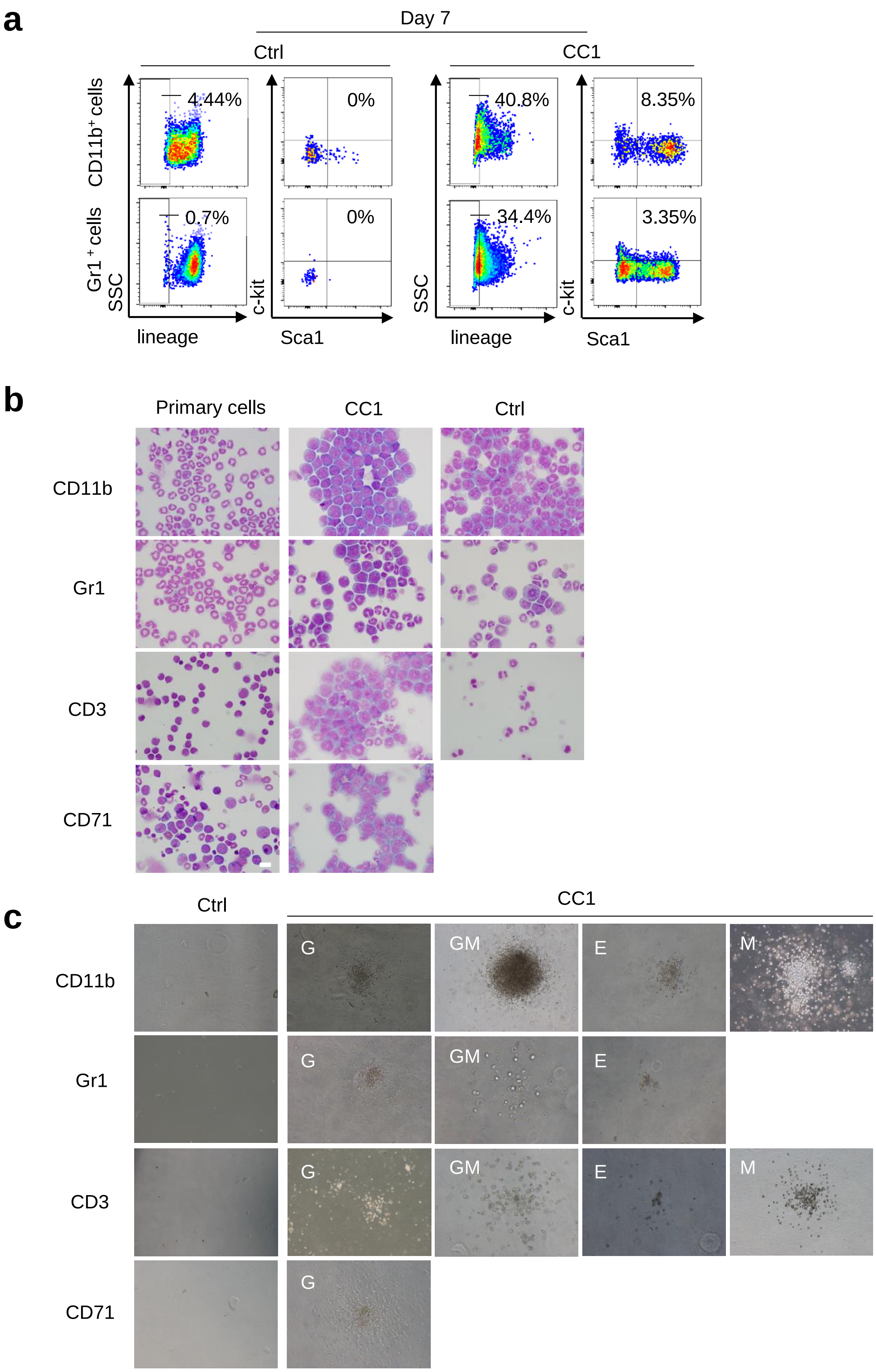
a
Day 7
Ctrl
CC1
CD11b+ cells
Gr1+ cells
4.44%
0%
40.8%
8.35%
0.7%
0%
34.4%
3.35%
SSC
c-kit
lineage
Sca1
b
Primary cells
CC1
Ctrl
CD11b
Gr1
CD3
CD71
c
Ctrl
CC1
CD11b
Gr1
CD3
CD71
G
GM
E
M
